# Basic Simulation Environment for Highly Customized Connected and Autonomous Vehicle Kinematic Scenarios

**DOI:** 10.3390/s17091938

**Published:** 2017-08-23

**Authors:** Linguo Chai, Baigen Cai, Wei ShangGuan, Jian Wang, Huashen Wang

**Affiliations:** 1 School of Electronics and Information Engineering, Beijing Jiaotong University, NO. 3 Shangyuancun, Haidian, Beijing 100044, China; lgchai@bjtu.edu.cn (L.C.); wangj@bjtu.edu.cn (J.W.); hswang@bjtu.edu.cn (H.W.); 2 School of Computer and Information Technology, Beijing Jiaotong University, Beijing 100044, China; bgcai@bjtu.edu.cn

**Keywords:** connected and autonomous vehicles, road network description, vehicle kinematic models, uncertainties modelling, application verification, simulation platform

## Abstract

To enhance the reality of Connected and Autonomous Vehicles (CAVs) kinematic simulation scenarios and to guarantee the accuracy and reliability of the verification, a four-layer CAVs kinematic simulation framework, which is composed with road network layer, vehicle operating layer, uncertainties modelling layer and demonstrating layer, is proposed in this paper. Properties of the intersections are defined to describe the road network. A target position based vehicle position updating method is designed to simulate such vehicle behaviors as lane changing and turning. Vehicle kinematic models are implemented to maintain the status of the vehicles when they are moving towards the target position. Priorities for individual vehicle control are authorized for different layers. Operation mechanisms of CAVs uncertainties, which are defined as position error and communication delay in this paper, are implemented in the simulation to enhance the reality of the simulation. A simulation platform is developed based on the proposed methodology. A comparison of simulated and theoretical vehicle delay has been analyzed to prove the validity and the creditability of the platform. The scenario of rear-end collision avoidance is conducted to verify the uncertainties operating mechanisms, and a slot-based intersections (SIs) control strategy is realized and verified in the simulation platform to show the supports of the platform to CAVs kinematic simulation and verification.

## 1. Introduction

Traffic simulation software is widely used in verifying and optimizing the traffic coordinating algorithms, due to its characteristics of repeatability, maneuverability, accuracy and low-cost. For the purpose of being aimed at different requirements, different traffic simulation software is developed. CORSIM, SimTraffic, AIMSUN, VISSIM, PARAMICS, etc., are mostly used in microscopic traffic simulation, while CORFLO, KRONOS, KWaves [1], etc., are used in macroscopic traffic simulation and SPSS, STATISTICA [2,3] are mostly used in vehicle dynamical analyzing. To better simulate different traffic scenarios, most of the software supports application program interfaces (APIs) are for secondary developing, and researchers have obtained achievements based on traffic simulation software.

As the development of sensor and communication technology improves, connected and autonomous vehicles (CAVs), connected vehicles (CVs) and autonomous vehicles (AVs) [4] are able to better serve traffic. Due to the advantages of simulation, many control strategies of CAVs have been conducted in simulation environment.

Gueriau [5] proposed a simulation framework by introducing cooperative traffic models based on Multi-Agent theory into an open-source traffic simulator. The resulting simulation framework is robust and able to assess potential benefits of cooperative traffic control strategies in different traffic configurations. Zha [6] proposed and evaluated a dilemma zone protection framework via vehicle to infrastructure communications, and examined the effectiveness of the proposed framework in a simulation test bed built in VISSIM. Fredette [7] developed a simulated road network to resolve the disconnect between powertrain engineering and eco-driving by presenting the development of a simplified methodology for ensuring that eco-driving scenarios are contextualized against traditional drive-cycle based text techniques. Goodall [8] proposed a novel technique to estimate the positions of unconnected vehicles based on the behaviors of communicating vehicles along a signalized arterial and simulated and verified the method microscopically using a commercial traffic simulation software package.

From the analysis above, we can conclude that models and control strategies verification can be grouped into three categories: the first category is API based verification in commercial traffic simulation software, such as Paramics and Vissim; the second category is redevelopment beyond open source traffic simulation software, such as MITSIMLab [9] and SUMO [10]; and the third category is to establish a new fundamental simulation environment.

The main difference between CAVs simulation and traditional traffic simulation is the controlling objects. In traditional traffic simulation, parameters of signal information, channelization, link properties, etc., are changed to enforce vehicles to change behaviors. In CAVs simulation, based on the V2X communication and status perception, control strategies should be generated to each individual vehicle to better simulate the autonomy of the vehicle. Due to the operation mechanism of the traffic simulation software, sometimes it is very complicated for researchers to implement customized models or control strategies within the API constraints, or models and algorithms we implement in the software are not able to operate as we anticipate. In this situation, fundamental simulation environment should be built to verify the models and the control strategies.

Control strategies of CAVs are usually generated based on status perception and communication. Radar [11], LIDAR, camera, GPS, etc., are equipped on the vehicle or mounted on the infrastructure to perceive vehicle and environment status. The purpose of perceiving vehicle and environment status is to precisely judge the spatial and temporal relationship between two traffic objects, so the decision-making component can generate an optimal control strategy and better coordinate the traffic. The perceived status can be transmitted by several kinds of communication modes, like DSRC [12], 4G, 3G, WIFI, etc. The performance of different communication modes have been deeply researched to help find appropriate communication mode applied in a certain traffic scenario [13]. In CAVs based scenarios and applications simulation, the accuracy of the perceived status and the performance of the communication should be taken into account to enhance the reality of the simulation.

In this paper, the framework, models and implementation of CAVs kinematic simulation and verification are discussed. A four-layer framework for CAVs kinematic simulation is proposed. Properties of the intersections are defined to describe the road network. A target position based vehicle position updating method is designed to restrict the vehicles to move within the links, as well as to simulate the behaviors of lane changing and turning. Vehicle kinematic models are implemented to maintain the status of the vehicles. Priorities for individual vehicle control are authorized to different layers. Operating mechanisms of the CAVs uncertainties, which are defined as position error and communication delay, are implemented in the simulation. A simulation platform based on the proposed methodology is developed, and experiments are conducted to prove the reliability of the methodology.

## 2. Multi-Layer Simulation Framework

**Figure 1:**
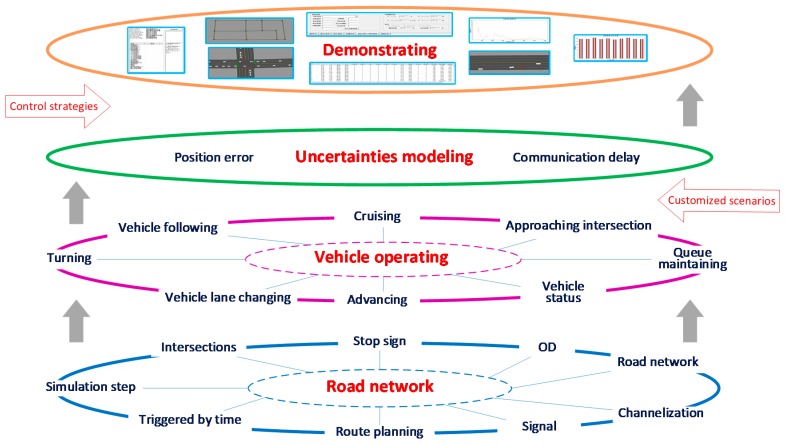
Framework of Connected and Autonomous Vehicles (CAVs) kinematic simulation.

A multi-layer framework is introduced to accomplish the simulation. Four layers are designed: road network layer, vehicle operating layer, uncertainties modelling layer and demonstrating layer. Relationship of each layer is showed in Figure 1.

The road network layer is to define the properties of the intersection and to describe the road network. Intersections are considered as the basic elements, links are generated based on the properties of each intersection. Signal control strategy and stop sign control strategy of intersections are deployed in road network layer. In vehicle operating layer, vehicles are generated from zone intersections, and move within the rules of vehicle following, lane changing, route selecting and other vehicle kinematic models. In CAVs, vehicle position error and communication delay always exist. Positioning error and communicating delay models are implemented in error modelling layer to enhance the reality of the simulation. The demonstrating layer is for visualization and evaluation.

Based on the CAVs kinematic simulation framework, one vehicle is able to move from its start zone to its destination zone along the pre-generated route. For CAVs applications, scenarios and control strategies would be implemented in the simulation, and several components would modify the status of the vehicles. Thus, priority of vehicle control is authorized to different layers to ensure the stability of the simulation. A hazardous over-speed scenario and pre-warning control strategy would be analyzed as an example to explain the vehicle control priorities. When one vehicle is generated in the road network, basic vehicle kinematic models, which are vehicle following model, lane changing model, route selecting model, turning model, etc., would generate vehicle status step by step to lead the vehicle moving to its destination intersection. When the scenario conditions are satisfied, the vehicle would be controlled by the scenario component. Meanwhile, the control strategy would monitor the status of the vehicle, and generate pre-warning suggestions to control the vehicle if necessary. After the pre-warning is relieved, the authority to control the vehicle would be given back to the vehicle kinematic models. Thus, control strategies, scenarios and basic vehicle kinematic models are authorized with the priority of high, medium and low respectively. Vehicle control component would realize its function by either changing the acceleration of the vehicle, or altering the vehicle trajectory.

When deploying CAV-based applications in reality, usually infrastructures should be built. As one of the most important components in CAV system, infrastructure apparently has a number of functions: transferring information, surveilling vehicle and environment status, making decisions, etc. For the functions of communicating and computing, there is no need to set up infrastructures in the simulation, because in the simulation, status of every individual unit can be obtained and computing would be executed automatically based on pre-designed algorithms. For the roadside sensors, which are aiming to surveil the vehicle and environment status, they can be simulated as scenario and control strategy. Surveillance radar would be analyzed as an example. Surveillance radars are usually distributed at high risk points, such as road-crossing, railroad crossing, and giving information to the vehicles about the presence of moving or still objects in a certain area. To simulate the function of surveillance radar, firstly, the operating mechanism of the radar should be modeled, and data type of the surveilling information should be designed based on the pre-existing simulation environment. Then a new programming object should be defined to maintain the information collected by the sensor in each simulation step. The programming object should include position of the moving or still object, length of detection capability, ID of vehicles within the detection range, etc. More attributes of the programming object can be designed based on different simulation goals. Then a control strategy, which is to process the information collected by the radar, should be designed and applied in the simulation. The output of the control strategy can be customized by the user. It can be either the speed suggestion to the vehicle, or lane suggestion to the vehicle. Lastly, we can evaluate the performance of the surveillance radar by analyzing the movement of the vehicles.

In this paper, properties of the vehicles and the intersections are defined in detail for vehicle control components to better control the vehicles, and a target position based vehicle position updating method is introduced to accomplish the change of vehicle trajectory.

## 3. Definition of the Properties of Intersections and Vehicles

### 3.1. Properties of the Intersection

Intersections are the basic elements composing the road network. Links can be described according to the properties of the intersections. In this paper, intersection properties are defined to describe a road network. The properties are defined in Table 1.

**Figure 2:**
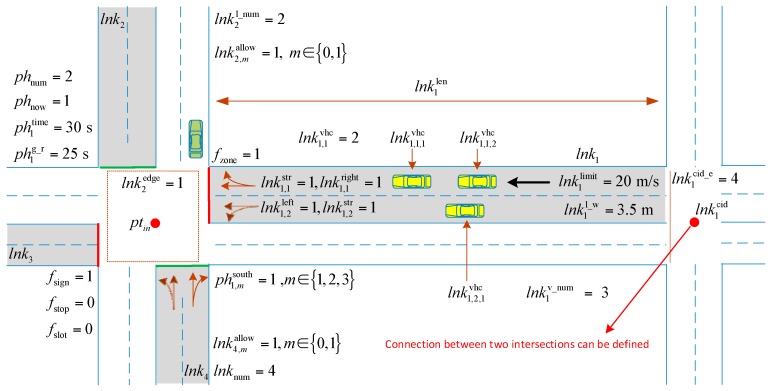
Properties of the intersection.

Each intersection would have corresponding properties and the properties are initialed before the simulation starts. After the simulation begins, the properties are updated in each simulation step. The column that is marked as fixed indicates that whether the value would change in each simulation step. The proposed method supports up to four directions of one intersection. The up edge of the intersection is figured as north, bottom edge figured as south, left edge figured as west, and right edge figured as east. In order to make the properties of intersections easier to understand, a road network with two intersections is introduced to explain the meanings of the properties in Figure 2.

Paper uses properties of intersection to define the properties of link. When one intersection is being placed in the simulation environment, the relationship of the intersection and the pre-selected intersection would be established automatically (connecting link exists). The links would automatically be updated if the positions of the intersections are changed, and the connecting edge of the link to the intersection would be updated. When the simulation is began, each intersection would have a corresponding programming object to maintain the status of the intersection by updating the parameters of the object in each simulation step. In this way, we can built enough large road network and guarantee the simulation reliability.

### 3.2. Properties of the Vehicle

The properties of vehicle are designed as below in Table 2.

**Figure 3:**
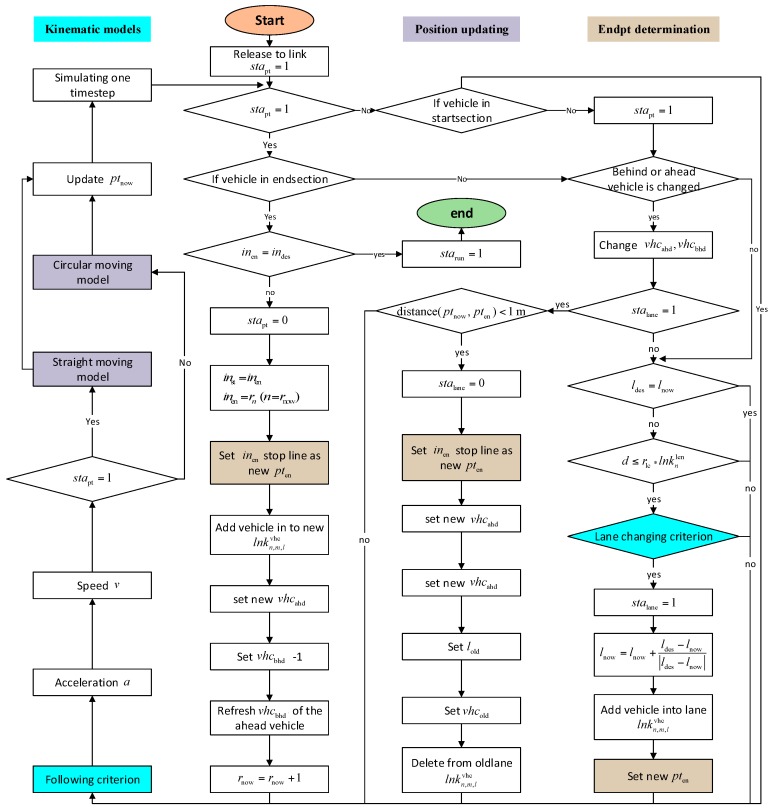
Vehicle operation process.

Same with the intersection properties, each vehicle would have corresponding properties. The properties would be initialed when the vehicle is released, and would be updated in each simulation step. The column that is marked as fixed indicates that whether the value would change in each simulation step. When one vehicle is released into the road network from zone intersections based on Origin Destination (OD) information, properties that contain route, lane, destination lane, initial speed, ahead vehicle, start intersection, end intersection, destination intersection, lane changing rate, etc., of the vehicle would be set to defaults. All the default values can be customized based on different requirements by the users. The route information is calculated based on a minimum route distance method [14], and other route selecting methods apply. Then the vehicle would move in a straight way towards the target position. Vehicle queues of each lane would be maintained rigorously to apply the vehicle following model. When the vehicle is changing lane or entering the intersection, the target position would be changed to simulate different vehicle behaviors. For the vehicle that is entering the intersection, if the vehicle is turning, a circular moving model [15] is implemented to update its position, else straight position updating method is applied. And if the intersection is the destination of the vehicle, $sta_{\mathrm{run}}$ of the vehicle would be set to 1 and the vehicle would not be updated or displayed in the simulation. Acceleration and the target position are the main attributes that are leading the vehicle. Acceleration is determined by vehicle following and cruising models, and the target position is determined by vehicle trajectory. To better explain how the vehicles are controlled by each component, Figure 3 is presented below to show the vehicle operation process from the time it is generated to the time it finishes its journey.

## 4. Maintaining of Vehicle Kinematic Status

### 4.1. Vehicle Speed Determination by Following and Cruising Models

**Figure 4:**
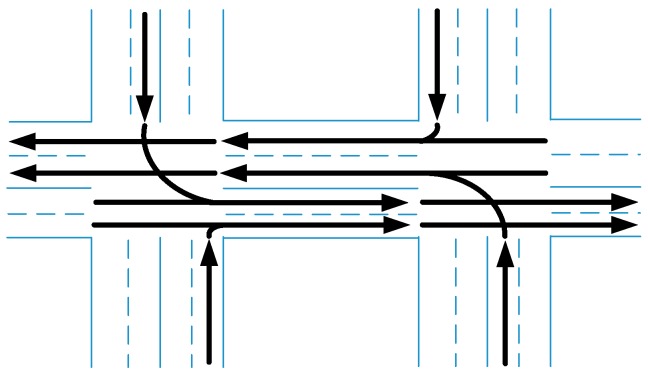
Segmented queue theory.

Movements of the following vehicle are significantly influenced by the leading vehicle. A segmented queue theory is applied to maintain the sequence of vehicles in each lane. The segmented queues are defined as below in Figure 4.

As shown in the figure, queues are defined based on the lanes. When one vehicle crosses the stop line of the intersection or change to another lane, the vehicle is involved into another queue and would be deleted from the previous queue. In some intersections, due to the channelization of the intersection, right turning vehicles and straight going vehicles may merge into the same queue. In this situation, the intersection control strategy would decide which to be the leading vehicle and which to be the following vehicle as the vehicles are prioritized to enter the intersection. The queue information is saved in $lnk_{n,m,l}^{\mathrm{vhc}}$ and updated as the simulation proceeds. Minimum safe distance vehicle following model [16] and constant speed cruising model are applied to determine the acceleration and speed of the vehicles. The vehicle following model can be described as below.

(1) $$s_{gap}+\frac{v_{l}^{2}}{2a_{dcc}}\geq \tau +\frac{v_{f}^{2}}{2\left(a_{dcc}/2\right)}$$

where $v_{l}$ denotes the leading vehicle speed, $v_{f}$ denotes the following vehicle speed, $a_{dcc}=6{\;m/s}^{2}$ is the maximum deceleration [17], $s_{gap}$ is the gap between the two vehicles, τ is the minimum safe distance between two vehicles with value of 5 m. Vehicle speed can be updated as below:

(2) $$\left\{ \begin{array}{l} v_{n+1}=v_{n}+a_{dcc}\times t_{step}/2\;(s_{gap}+{v_{l}}^{2}/\left(2a_{dcc}\right)\geq \tau +{v_{f}}^{2}/a_{dcc}\;\&\;v_{n}<v_{limit})\\ v_{n+1}=v_{n}\;(s_{gap}+{v_{l}}^{2}/\left(2a_{dcc}\right)\geq \tau +{v_{f}}^{2}/a_{dcc}\;\&\;v_{n}\geq v_{limit})\\ v_{n+1}=v_{n}-a_{dcc}\times t_{step}/2\;(s_{gap}+{v_{l}}^{2}/\left(2a_{dcc}\right)<\tau +{v_{f}}^{2}/a_{dcc}) \end{array}\right.$$

$v_{n}$ denotes the vehicle speed in time step *n*, and $v_{n+1}$ denotes the vehicle speed in time step *n* + 1, $v_{limit}$ denotes the link speed limit, $t_{step}$ is the interval of time step, usually the range of $t_{step}$ is from 10 ms to 1 s [18]. When one vehicle is approaching the intersection stop line, the vehicle should stop if the wayleave of the lane is not authorized. The wayleave of the lane is determined by the attributes of signal and channelization.

### 4.2. Vehicle Position Updating Method

**Figure 5:**
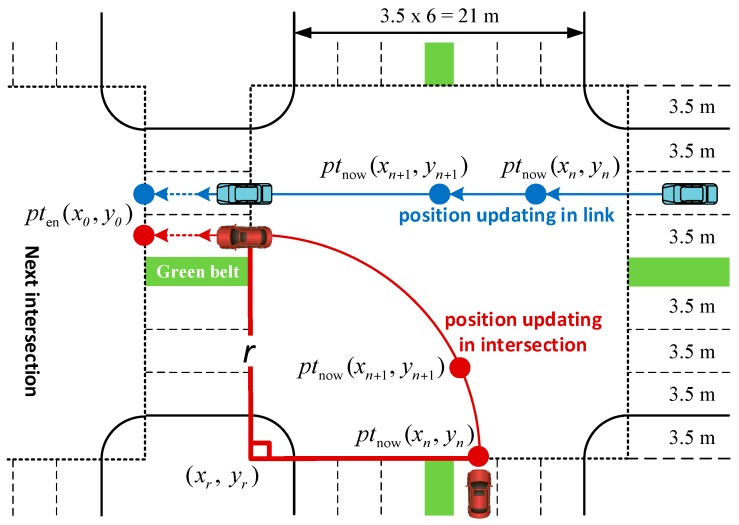
Position updating.

Figure 5 shows the vehicle position updating processes of straight going and turning.

$\left(x_{n},y_{n}\right)$ denotes the position of the vehicle at simulation step n, $\left(x_{0},y_{0}\right)$ denotes the $pt_{\mathrm{en}}$ at simulation step n, $\left(x_{n+1},\;y_{n+1}\right)$ denotes the position of the vehicle at simulation step n+1, r denotes the vehicle turning radius which can be calculated with the properties of the intersection, $\left(x_{r},\;y_{r}\right)$ denotes the center of the turning circle, $\left(x_{n+1},\;y_{n+1}\right)$ is calculated within the criteria of vehicle kinematic models and trajectories. For turning vehicles, $\left(x_{n+1},\;y_{n+1}\right)$ can be calculated by the formula below.

(3) $$\left\{ \begin{array}{l} \theta =t_{step}\times v/r+\arcsin((y_{n}-y_{r})/r)\\ x_{n+1}=x_{r}+r\times \cos\theta \\ y_{n+1}=y_{r}+r\times \sin\theta \end{array}\right.$$

For the straight going vehicles, $\left(x_{n+1},\;y_{n+1}\right)$ can be calculated by the formula below.

(4) $$\left\{ \begin{array}{l} \left(x_{n+1}-x_{n})/(y_{n+1}-y_{n})=(x_{0}-x_{n})/(y_{0}-y_{n}\right)\\ {\left(x_{n+1}-x_{n}\right)}^{2}+{\left(y_{n+1}-y_{n}\right)}^{2}={\left(v\times t_{\mathrm{step}}\right)}^{2} \end{array}\right.\;\left(\begin{array}{c} y_{n}<x_{n+1}<x_{0}\\ y_{n}<y_{n+1}<y_{0} \end{array}\right)$$

As showed in formula, updating of the position of the turning vehicle does not rely on $pt_{\mathrm{en}}$ but based on the properties of the intersection instead. However, straight going vehicle position updating should relate to $pt_{\mathrm{en}}$. A target position maintaining method is proposed to lead the vehicle to move along its trajectory.

### 4.3. Target Position Updating

**Figure 6:**
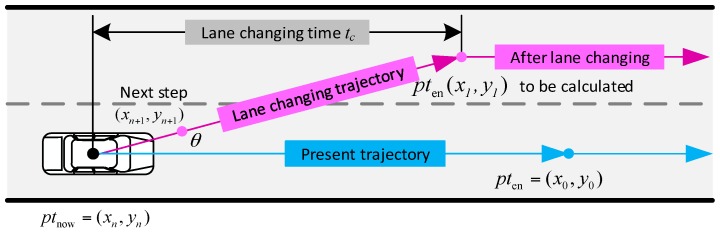
Target position redefined when vehicle is changing lane.

There are two situations that the target position of the vehicle should be modified. One is when the vehicle is changing lane, and other is when the vehicle is entering the intersection. The inequation below shows a minimum safety distance based vehicle lane changing model criterion implemented in the platform.

(5) $$s_{lon}\geq s_{f}-s_{l}+L+W\times \sin\theta$$

where $s_{lon}$ is the longitudinal gap between the lane changing vehicle and its behind vehicle in the target lane, $s_{f}$ is the longitudinal distance the behind vehicle has moved during the lane changing duration $t_{c}$, $s_{l}$ is the longitudinal distance the lane changing vehicle has moved during the lane changing duration $t_{c}$. In this paper, $t_{c}$ is set to 3s [19]. L is the length of the vehicle, W is the width of the vehicle, θ denotes the angle between the lane and the direction that the lane changing vehicle moves to. When the attribute $r_{\mathrm{lc}}$ and the minimum safety distance based lane changing criterion are satisfied, vehicle starts to change lane, the target position can be calculated in Figure 6.

By solving the equations below, the target position can be obtained.

(6) $$\begin{array}{l} syms\;x_{1}\in (x_{n},x_{0}),\;y_{1}\in (0,\infty )\\ \left\{ \begin{array}{l} {\left(x_{1}-x_{n}\right)}^{2}+(y_{1}-y_{n}{)}^{2}=(v_{n}t_{c}{)}^{2}\\ \frac{\left|(y_{0}-y_{n})x_{1}-(x_{0}-x_{n})y_{1}+x_{0}y_{n}-y_{0}x_{n}\right|}{sqrt({\left(y_{0}-y_{n}\right)}^{2}+{\left(x_{0}-x_{n}\right)}^{2}}=w_{l} \end{array}\right. \end{array}$$

When one vehicle finishes lane changing movement, or enters the intersection, the new target position would be set to the middle of stop line of the present lane. Except for the lane changing triggering condition, the target position should be updated when the distance between the vehicle and the target position is smaller than 1 m.

## 5. Uncertainties Operating Mechanisms

**Figure 7:**
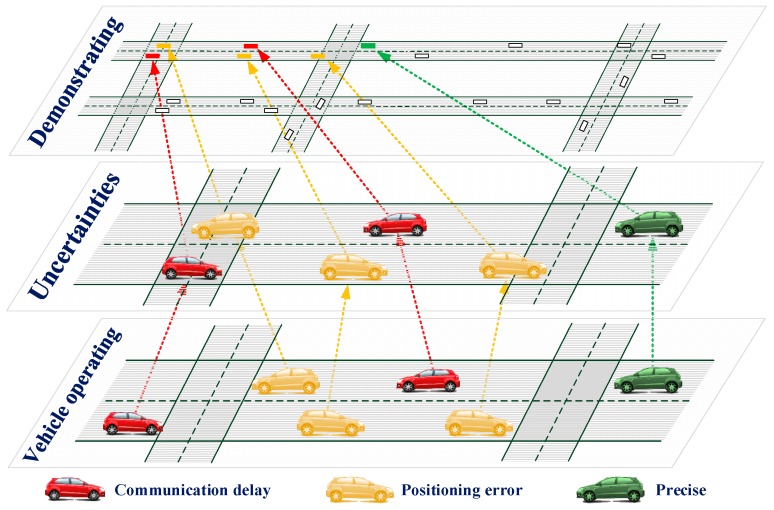
Observing positions of vehicles influenced by uncertainties.

Qualities of communication and positioning would significantly influence the effectiveness of applied algorithms and control strategies. Position error and communication delay models and operating mechanisms are analyzed in this paper. Figure 7 shows the observing position of the vehicle that is influenced by the uncertainties in vehicle operating layer, uncertainties modelling layer and the demonstrating layer.

As shown in the figure, in the vehicle operating layer, the vehicles are moving according to the vehicle kinematic models. In the uncertainties modelling layer, the observed positions of the vehicles are changed due to the overlapped position error and the communication delay. The demonstrating layer is for displaying and the display of the vehicles and the road network can be scaled and translated. Lateral position error mainly effects the lane identification of the vehicle. Now this problem can be well resolved by real-time video processing technology with accuracy of 97% [20]. In this case, only longitudinal position error is discussed. Communication delay would influence the punctuality of information transmitting. Frequent information interacting would significantly influence the performance of the system. A CAV system contains variety of communication interacting types: interaction within one single device, transmission between two different equipment, multi-hop transmission among several components. Different communication protocols would lead to different communication delays. In this paper, single-hop transmission is discussed as an example for modelling the communication delay. Other communication delay models can be applied by replace the proposed model in this paper. Longitudinal position error and communication delay would affect the observing distance between two vehicles. In this case, it may lead to false pre-warning or pre-warning failure if no filtering algorithms are taken.

### 5.1. Gaussian Distribution Based Position Error Model

It is difficult to establish an accurate error model for the position error; nowadays, most existing methods are based on Gaussian distribution. Lee [21] defined Gaussian distribution-based trajectory models to design the threshold parameters for a rear-end collision avoidance system. Although some researchers use other methods to model the GPS positioning error as a practical alternative [22], we still propose the mostly adopted bounding method (Gaussian distribution function) to represent the distribution of position error φ.

(7) $$F(\varphi )=\int_{-\infty }^{\varphi }\frac{1}{\sqrt{2\pi \sigma^{2}}}e^{-{\left(y-\mu \right)}^{2}/2\sigma^{2}}dy$$

**Figure 8:**
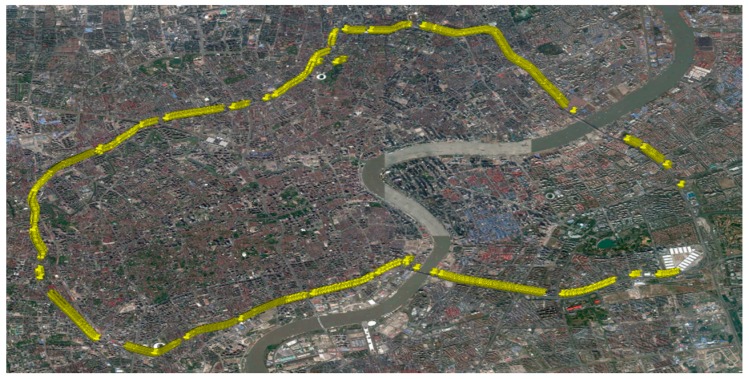
Vehicle trajectory of the experiment.

**Figure 9:**
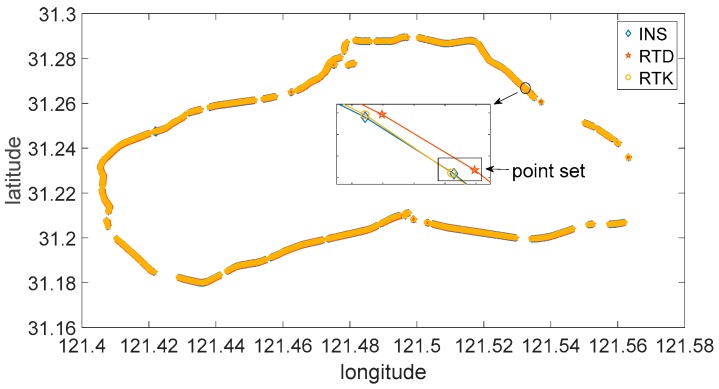
Data map and point set.

To determine the value of σ and μ in the above equation, an experiment has been conducted in Shanghai, China. A car equipped with three kinds of positioning systems are driven on the express way to generate positioning data. The three kinds of positioning systems are: INS (Inertial Navigation System) with RTK (Real Time Kinematic) positioning (this system is considered as the most precise system in the experiment, the other two systems are compared with this one), RTK positioning system and RTD (Real Time Differential) positioning system. The trajectory of the experiment is showed in Figure 8 and Figure 9.

**Figure 10:**
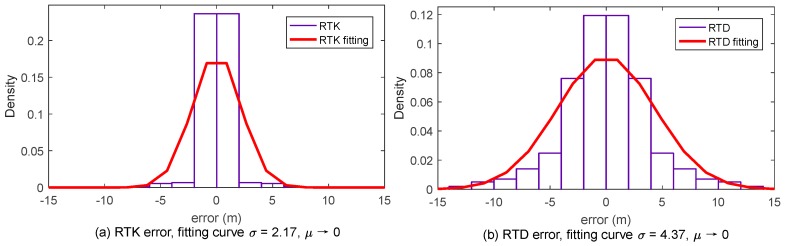
Density of the RTK and RTD position error: (**a**) density of RTK positioning error and its fitting curve; and (**b**) density of RTD positioning error and its fitting curve.

Positioning data is collected once per second in each positioning system. Invalid point sets (at a certain time, one or more positioning systems did not generate valid position data) are deleted (the discontinuity of the trajectory is because of this) from the data sheet. Total number of the valid point sets is 4728. Position error is defined as the distance between INS position and RTK/RTD position. So we can get the position error matrix $e_{k}$ and $e_{d}$. $e_{k}$ denotes the error of RTK positioning system, and $e_{d}$ denotes the of RTD positioning system. $e_{k}$ and $e_{d}$ are both 1 × 4728 matrix. With the definition of $e_{k}$ and $e_{d}$, we can see that all the values in $e_{k}$ and $e_{d}$ are positive. Considering that the mean value of positioning error should approximates to 0 and the symmetry of position error, we establish new position error matrix $\left[-e_{k}\;e_{k}\right]$ and $\left[-e_{d}\;e_{d}\right]$ for analyzing. The densities of RTK and RTD position error are showed in Figure 10.

The position error distribution is subject to Gaussian distribution. RTK positioning system is more precise than RTD positioning system, so σ is smaller in RTK position error density fitting curve. In fact, σ differs in different positioning systems and different environments. Dion [23] has established a position error model with σ equals to 2.8. In this paper, we use the RTD error model. Position error complying with the distribution above is superposed on each vehicle.

### 5.2. Uniform and Rayleigh Distribution-Based Communication Delay Model

**Figure 11:**
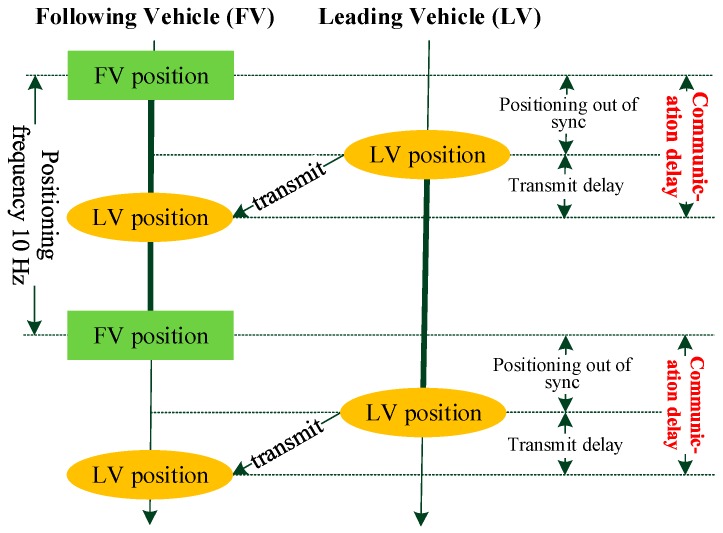
Communication delay generating process.

Due to different system structures and information retransmission mechanisms, communication delay is difficult to evaluate. For V2V communication, DSRC is widely used. In this paper, DSRC-based single-hop V2V communication delay is discussed. The communication delay generating process can be described in Figure 11.

Generally, positioning devices output vehicle position data 10 times per second. The positioning time of the following vehicle and the leading vehicle cannot be accurately synchronized. This kind of asynchronization leads to communication delay, and the information transmission also results in communication delay. For the part of communication delay $d_{1}$ caused by the asynchronization of positioning time, uniform distribution is applied to describe it. The distribution function can be defined as below.

(8) $$F_{1}(d_{1})=\int_{0}^{d_{1}}\frac{1}{100}dy$$

The transmission delay model is established based on the data in paper [23]. After analyzing the data in fiducial probability of 0.5, we found that the data is subject to Rayleigh distribution with mean value equals to 29.98 ms. Probability density function of Rayleigh distribution is defined as below.

(9) $$f_{2}(x)=\frac{x}{\sigma^{2}}e^{-x^{2}/2\sigma^{2}}$$

And due to the characters of Rayleigh distribution, the mean value is defined as below.

(10) $$E(D_{2})=\sigma \sqrt{\pi /2}\approx 1.253\sigma =29.98$$

Then we can get of value of σ=23.93. With Equation (9), we can get the transmission delay model as below.

(11) $$F_{2}(d_{2})=\int_{0}^{d_{2}}\frac{y}{572.6}e^{-y^{2}/1145.3}\;dy$$

**Figure 12:**
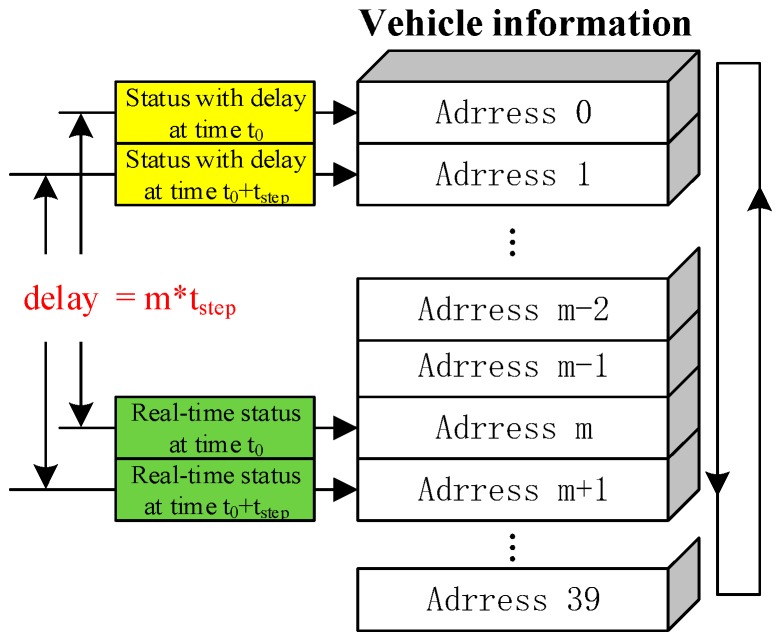
Communication delay simulating process.

The communication delay is the sum of asynchronization delay and transmission delay. To implement the communication delay model in simulation, an array with 40 rows has been defined in computer memory. The communication delay simulating process is showed in Figure 12.

Vehicle information would be saved in the array step by step. An index is used to determine which item should be used to be displayed or to be applied in the applications. For other communication modes, the communication delay models are able to be applied in the simulation within the proposed framework.

## 6. Simulation and Verification

**Figure 13:**
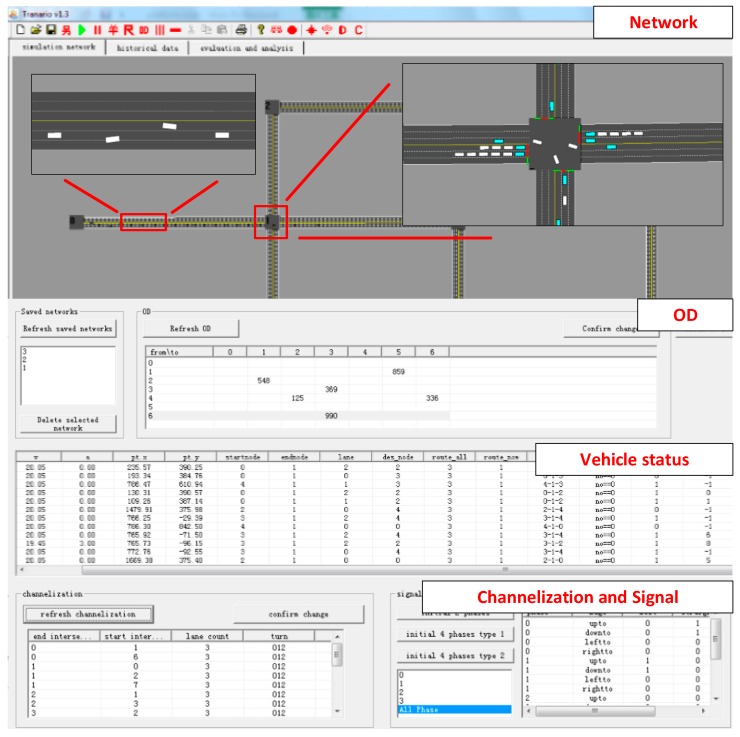
Simulation platform.

Besides the proposed simulation method, a user interface with a dozen of functions, which include establishing road network, setting parameters of the input, setting consecutive simulation model or single step model, road network saving, illustrating the simulation with graphics device interface (GDI) or GDI+, scaling and translating the display, etc., is designed to enhance the simulation. The simulation platform is showed in Figure 13.

The comparison of link delay to traditional road network, influence of uncertainties to the pre-warning algorithms and a CAVs application are conducted in the platform to verify the applicability and reliability of the platform.

### 6.1. Vehicle Delay Comparison with Traditional Road Network

**Figure 14:**
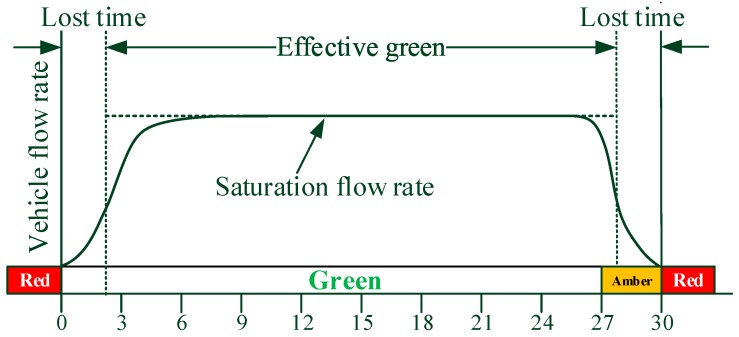
Deterministic queuing analysis.

Scenarios and applications of CAVs are all based on traditional traffic flows. That whether the basic traffic flow coincides with the real traffic flow would significantly influence the results of any control strategies and algorithms verified in the platform. Vehicle average delay is analyzed in condition of fixed signal control to test and verify the reality of the platform. Volume to capacity ratio is introduced to the verification. The volume to capacity ratio is defined as below.

(12) $$\eta =\frac{\varphi }{\phi g_{e}/C}$$

where φ denotes the vehicle arrival flow rate, ϕ denotes the saturation flow rate, C denotes the traffic signal cycle length, $g_{e}$ denotes the effective green interval duration, and η denotes the volume to capacity ratio. The saturation flow rate value equals to 1800 vhc/h [24]. The saturation flow rate is defined in Figure 14.

**Figure 15:**
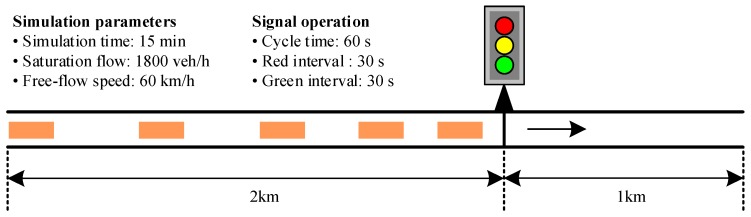
Delay evaluation scenario.

Due to the 1994 HCM delay estimate model [25], a one-lane-one-intersection scenario is set up and simulated for 15 min to estimate the delay. The scenario is showed in Figure 15.

**Figure 16:**
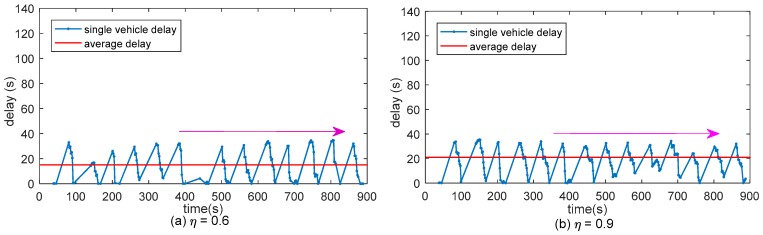

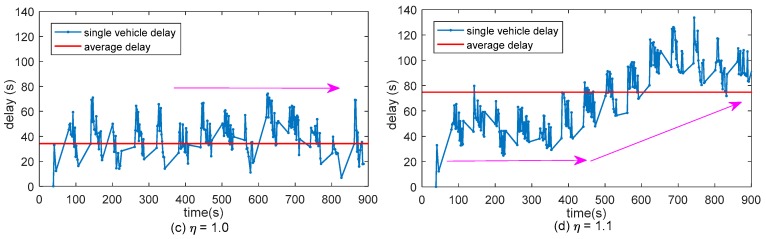
Vehicle delay in different volume to capacity ratio: (**a**–**d**) are the vehicle delay when η equals to 0.6, 0.9, 1.0 and 1.1 respectively.

Thanks to the vehicle kinematic models implemented in the platform, the saturation flow is approximately 1800 vhc/h at signal intersection. Figure 16 shows the results of the delay of every vehicle and the vehicle average delay in different volume to capacity ratio.

**Figure 17:**
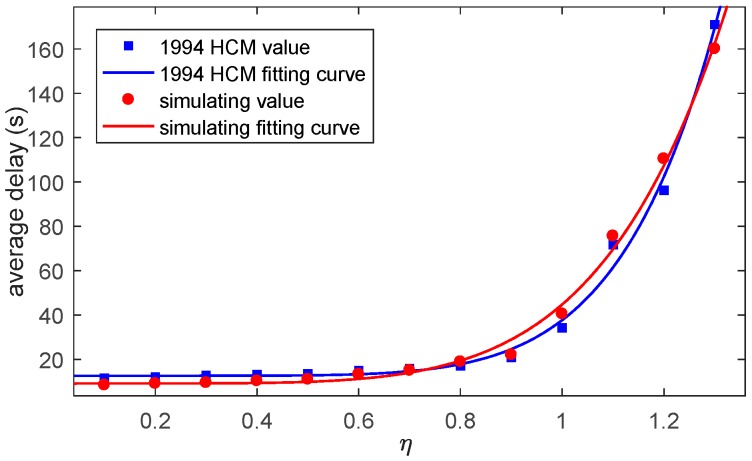
Average delay in different conditions.

Delay data is calculated and saved when one vehicle reached its destination. Single vehicle delay vibrates because some of the vehicles are influenced by the red light while some vehicles are not. Average vehicle delay rises along with η. When η≤1, single vehicle delay value remains in a certain range and when η>1 single vehicle delay is rising along with the time, this condition reflects the definition of volume to capacity ration. Average delay in different η is simulated and is showed in Figure 17.

Compared to the results from Dion [24], the results simulated by the platform correspond to the results calculated by the HCM delay model, which indicate that the platform is reliable.

### 6.2. Verification of the Uncertainties

**Figure 18:**
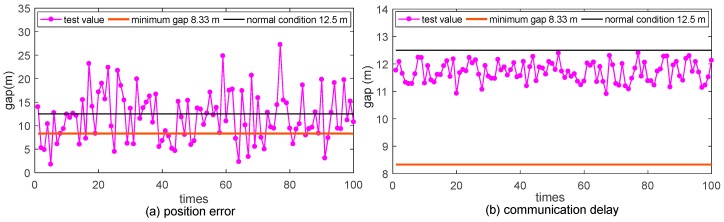
Verification of uncertainties: (**a**) gap between two vehicles when applying the Gaussian distribution positioning error model; (**b**) gap between two vehicles when applying the uniform and Rayleigh distribution communication delay model.

Pre-warnings are adopted to analyze the effect of the uncertainties models implemented in the platform. Hazardous vehicle maneuver of over speed is generated as basic scenario, and braking distance based pre-warning method with maximum deceleration of 6 m/s^2^ [26] is embedded as the control strategy. The aforementioned Gaussian distribution based position error model with standard deviation of 4.37 and average value of 0, and Rayleigh distribution based DSRC communication delay model with standard deviation of 23.93 and average value of 29.98 are applied to simulation the uncertainties. The speed of the following vehicle is 30 m/s and the speed of the leading vehicle is 20 m/s. The scenario was executed 100 times and the results are showed in Figure 18.

In the figure, the Y-axis denotes the distance between the following vehicle and the leading vehicle when the pre-warning is generated. The results of test value line are based on the test of uncertainties. The minimum gap line is based on the condition that the deceleration value in warning method equals to 6 m/s^2^ and the normal condition line is based on the condition that the deceleration value in warning method equals to 4 m/s^2^. In position error and communication delay simulation, the results fluctuate around the theoretical result. The distribution of the results corresponds with the Gaussian distribution model and Rayleigh distribution model. The results proved the reliability of the platform.

### 6.3. Verification of CAVs Application: Slot-Based Intersections

**Figure 19:**
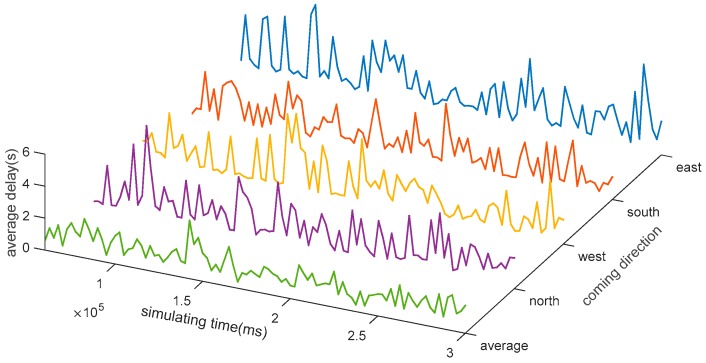
Average delay of each link in Sis.

Tachet [27] proposed a slot-based intersections (SIs) control strategy in CAVs. The concept of SIs is to enlarge the distance between two vehicles deliberately so that the conflicting vehicle can cross the intersection within the gap between the two vehicles. The SIs control strategy is implemented in the simulation platform and the control strategy is realized between the demonstrating layer and the uncertainties modeling layer. The intersection properties are the same with Figure 5. Simulation of input of 360 vhc/h of each lane has been conducted and the average delay of the vehicles is showed in Figure 19.

**Figure 20:**
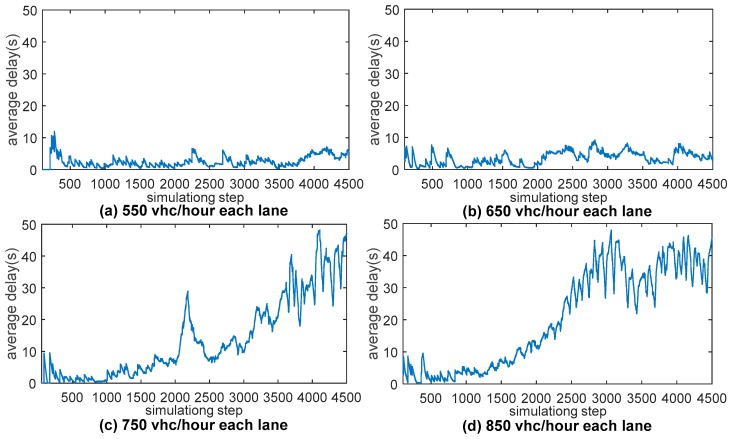
Average delay in different inputs: (**a**–**d**) are average vehicle delay when the input of each lane is 550, 650, 750 and 850 vhc/h respectively.

The coming directions are defined in the properties of intersection. Furthermore, based on the control strategy, simulation with input of 550, 650, 750 and 850 vhc/h per each lane has been conducted to estimate the bottleneck of the method. The results are showed in Figure 20.

**Figure 21:**
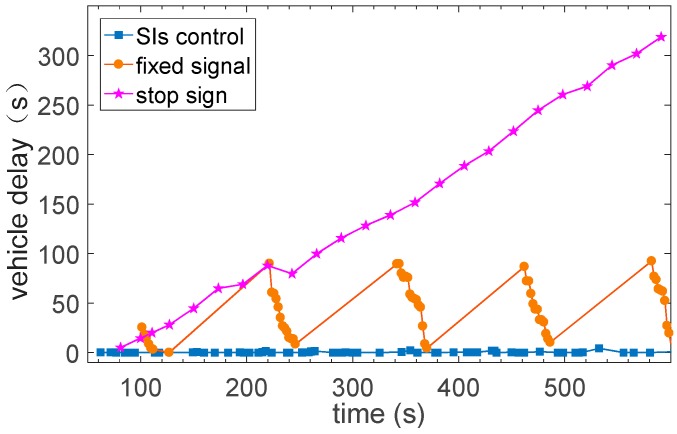
Comparison among different control strategies.

The bottleneck of the SIs control strategy does exist, and the value is between 650 and 750 vhc/h per lane. The simulation and the results indicate that the simulation platform is able to support the simulation and verification of CAVs applications. And three different intersection control strategies that are signal control, stop sign control and SIs control, are compared in the platform. And the simulation results are consistent with Tachet [27]. The results are showed in Figure 21.

## 7. Conclusions

A four-layer framework, which is composed with road network layer, vehicle operating layer, uncertainties modelling layer and demonstrating layer, for CAVs kinematic simulation is proposed in this paper. By conducting verification of comparison of link delay to traditional road network, influence of uncertainties to the pre-warning algorithms and a CAVs application, the methodology proposed in this paper turns out to be reliable. This paper proposed a method of how to establish the basic CAVs kinematic simulation environment. Framework and models are discussed. The correlations of different vehicle kinematic models, vehicle trajectory and road network are established. Based on the basic CAVs simulation environment, highly specialized scenarios and control strategies related to CAVs are able to be realized precisely. Due to the four layer framework of the methodology, models are easy to be replaced or updated as long as the outputs of the new models are subject to the definitions of the intersections and the vehicles.

In the proposed method, the number of maximum directions of an intersection is designed to be four. Although a four-legged intersection can cover most of the realistic situations, the intersection attributes still should be updated to involve more intersection characteristics. For different intersections, the in-intersection trajectory of the vehicle differs. The vehicle position updating method is not precise enough to describe the movement of the vehicles, especially in the situation when vehicle turns.

Infrastructures are not modeled and simulated in the paper. Although infrastructure can be simulated by separating its functions to scenario and control strategy, this is not an optimal solution. Infrastructure should be considered as an embedded component of the simulation framework. Basic operating mechanism of the infrastructure should be designed, and basic attributes of the infrastructure should be maintained.

This work proposed a method to build basic CAV kinematic simulation environment. The aim of the work is to help verify CAV based applications, so the performance of the simulation would directly influence the time-consuming of the verification. The simulation platform in this paper is established based on basic programming technology. The simulation would not be smooth enough when the road network and the traffic is large. Software engineering of computing, display based on GDI and the synchronization between them should be deeply researched. More uncertainties models should be implemented in the uncertainties modelling layer.

## Figures and Tables

**Table 1:** Definitions of intersection properties.

Name			Details	Fixed
**Basic**	$f_{\mathrm{slot}}$		The value equals 1 if the intersection is slot based intersection (SIs)	Yes
	$f_{\mathrm{stop}}$		The value equals 1 if the intersection is stop sign based intersection	Yes
	$f_{\mathrm{zone}}$		The value equals 1 if vehicles can be generated and ended in this intersection	Yes
	$f_{\mathrm{sign}}$		The value equals 1 if the intersection is signalized	Yes
	${pt}_{in}$		Position of the intersection center	Yes
**Link**	$lnk_{n}$		Index of the nth link	Yes
	$lnk_{\mathrm{num}}$		Count of links connected to this intersection	Yes
	$lnk_{n}^{\mathrm{cid}}$		Intersection ID at the other side of the $lnk_{n}$	Yes
	$lnk_{n}^{l\_\mathrm{num}}$		Count of lanes of $lnk_{n}$	Yes
	$lnk_{n}^{l\_w}$		Lane width of $lnk_{n}$	Yes
	$lnk_{n}^{\mathrm{edge}}$		Direction of $lnk_{n}$ to intersection A	Yes
	$lnk_{n}^{v\_\mathrm{num}}$		Count of vehicles in $lnk_{n}$ at a certain simulating step	No
	$lnk_{n}^{\mathrm{len}}$		Link length of $lnk_{n}$	Yes
	**Lane**	$lnk_{n,m}^{\mathrm{left}}$	The value equals 1 if lane m is authorized for left turning	Yes
		$lnk_{n,m}^{\mathrm{str}}$	The value equals 1 if lane m is authorized for straight going	Yes
		$lnk_{n,m}^{\mathrm{right}}$	The value equals 1 if lane m is authorized for right turning	Yes
		$lnk_{n,m}^{\mathrm{allow}}$	Determined by real-time signalization and channelization. If the value equals 1, vehicle is allowed to enter the intersection within lane m	No
		$lnk_{n,m}^{\mathrm{vhc}}$	Count of vehicles within lane m	No
		$lnk_{n,m,l}^{\mathrm{vhc}}$	ID of the vehicle which is queued lth within lane m in $lnk_{n}$	No
	$lnk_{n}^{\mathrm{limit}}$		Speed limit of $lnk_{n}$	Yes
	$lnk_{n}^{\mathrm{cid}\_e}$		Direction of $lnk_{n}$ to $lnk_{n}^{cid}$	Yes
**Signalization**	${ph}_{n}$		Index of the nth phase	Yes
	$ph_{\mathrm{num}}$		Count of signal phases	Yes
	$ph_{n,m}^{\mathrm{north}}$		m∈{0,1,2}, and 0, 1, 2 represent left turning, straight going and right turning respectively. If $ph_{n,m}^{\mathrm{north}}$ equals 1, the corresponding movement is authorized for the vehicle coming from north.	No
	$ph_{n,m}^{\mathrm{south}}$		See $ph_{n,m}^{\mathrm{north}}$	No
	$ph_{n,m}^{\mathrm{east}}$		See $ph_{n,m}^{\mathrm{north}}$	No
	$ph_{n,m}^{\mathrm{west}}$		See $ph_{n,m}^{\mathrm{north}}$	No
	$ph_{n}^{\mathrm{time}}$		Duration of phase n	Yes
	$ph_{n}^{g\_r}$		The remaining green time of phase n	No
	$ph_{\mathrm{now}}$		Index of the phase being executed	No

**Table 2:** Definitions of vehicle properties.

Name	Details	Fixed
$pt_{\mathrm{now}}$	Present position in a certain simulation step	No
$pt_{\mathrm{en}}$	Position that the vehicle is moving to	No
v	Speed of the vehicle	No
$in_{\mathrm{st}}$	Previous intersection id	No
$in_{\mathrm{en}}$	Next intersection id	No
$l_{\mathrm{now}}$	Index of the lane vehicle is now in	No
$sta_{\mathrm{pt}}$	The value equals 1 if the vehicle is in link	No
$in_{\mathrm{des}}$	Destination intersection	Yes
$r_{n}$	ID of the nth intersection vehicle should travel through	Yes
$r_{\mathrm{now}}$	Count of intersections vehicle has already passed	No
$sta_{\mathrm{run}}$	The value equals 1 if the vehicle reaches its destination intersection	No
$sta_{\mathrm{stop}}$	The value equals 1, vehicle stops	Yes
d	Distance from $pt_{\mathrm{now}}$ to $pt_{\mathrm{en}}$	Yes
$l_{\mathrm{des}}$	Destination lane, determined by route and channelization	Yes
$r_{\mathrm{num}}$	Count of intersections included in $r_{n}$	Yes
$vhc_{\mathrm{ahd}}$	ID of the vehicle ahead. The value equals −1 if no ahead vehicle exists	No
$vhc_{\mathrm{bhd}}$	ID of the vehicle behind. The value equals −1 if no behind vehicle exists	No
$sta_{\mathrm{lane}}$	The value equals 1 if vehicle is changing lane	No
$l_{\mathrm{old}}$	Previous lane	No
a	Acceleration of the vehicle	No
$vhc_{\mathrm{old}}$	Previous behind vehicle ID	No
$sta_{\mathrm{sc}}$	The value equals 1 if vehicle is involved in scenarios	No
$t_{\mathrm{re}}$	Driver’s reacting time when vehicle brakes	Yes
$vhc_{\mathrm{type}}$	Vehicle type, bus, truck or car	Yes
$r_{\mathrm{lc}}$	Determines when to change lane in a link	Yes
$sta_{\mathrm{slot}}$	The value equals 1 if the vehicle is controlled by slot based algorithm	No
$t_{\mathrm{stop}}$	Waiting time when vehicle is in a stop sign intersection	No
$sta_{\mathrm{stop}}$	The value equals to 1 if vehicle is stopped by stop sign intersection	No

## References

[1] Ratrout N.T., Rahman S.M. (2009). A comparative analysis of currently used microscopic and macroscopic traffic simulation software. Arabian J. Sci. Eng..

[2] Garg A., Vijayaraghavan V., Zhang J., Li S., Liang X. (2017). Design of robust battery capacity model for electric vehicle by incorporation of uncertainties. Int. J. Energy Res..

[3] Garg A., Chen F., Jian Z. State-of-the-art of designs studies for batteries packs of electric vehicles. Proceedings of the IET International Conference on Intelligent and Connected Vehicles (ICV 2016), Stevenage, UK, 22–23 September 2016.

[4] Mahmassani H.S. (2016). Autonomous vehicles and connected vehicle systems: Flow and operations considerations. Transp. Sci..

[5] Gueriau M., Billot R., El Faouzi N., Monteil J., Armetta F., Hassas S. (2016). How to assess the benefits of connected vehicles? A simulation framework for the design of cooperative traffic management strategies. Transp. Res. Part C Emerg. Technol..

[6] Zha L., Zhang Y., Songchitruksa P., Middleton D.R. (2016). An integrated dilemma zone protection system using connected vehicle technology. IEEE Trans. Intell. Transp. Syst..

[7] Fredette D., Pavlich C., Ozguner U. (2015). Development of a UDDS-Comparable framework for the assessment of connected and automated vehicle fuel saving techniques. IFAC PapersOnLine.

[8] Goodall N.J., Park B.B., Smith B.L. (2014). Microscopic estimation of arterial vehicle positions in a Low-Penetration-Rate connected vehicle environment. J. Transp. Eng..

[9] Du L., Ukkusuri S., Del Valle W.F.Y., Kalyanaraman S. (2009). Optimization models to characterize the broadcast capacity of vehicular ad hoc networks. Transp. Res. Part C Emerg. Technol..

[10] Griggs W.M., Ordonez-Hurtado R.H., Crisostomi E., Haeusler F., Massow K., Shorten R.N. (2015). A Large-Scale SUMO-Based emulation platform. IEEE Trans. Intell. Transp. Syst..

[11] Saponara S., Neri B. (2017). Radar sensor signal acquisition and multidimensional FFT processing for surveillance applications in transport systems. IEEE Trans. Instrum. Meas..

[12] Cheng L., Henty B.E., Stancil D.D., Bai F., Mudalige P. (2007). Mobile vehicle-to-vehicle narrow-band channel, measurement and characterization of the 5.9 GHz dedicated short range communication (DSRC) frequency band. IEEE J. Sel. Areas Commun..

[13] Saponara S., Giannetti F., Neri B., Anastasi G. (2017). Exploiting mm-Wave communications to boost the performance of industrial wireless networks. IEEE Trans. Ind. Inform..

[14] Shafiee K., Leung V.C.M. (2011). Connectivity-aware minimum-delay geographic routing with vehicle tracking in VANETs. Ad Hoc Netw..

[15] Leng J., Feng Y., Zhai J., Bao L., He Y. (2012). Travel time model of Left-Turning vehicles at signalized intersection. Math. Probl. Eng..

[16] Qu D., Chen X., Yang W., Bian X. (2014). Modeling of Car-Following required safe distance based on molecular dynamics. Math. Probl. Eng..

[17] Jiang R., Wu Q.S., Zhu Z.J. (2001). Full velocity difference model for a car-following theory. Phys. Rev. E.

[18] Abdelgawad K., Henning S., Biemelt P., Gausemeier S., Traechtler A. (2016). Advanced traffic simulation framework for networked driving simulators. IFAC PapersOnLine.

[19] Sheu J.B., Ritchie S.G. (2001). Stochastic modeling and real-time prediction of vehicular lane-changing behavior. Transp. Res. Part B Methodol..

[20] Gaikwad V., Lokhande S. (2015). Lane departure identification for advanced driver assistance. IEEE Trans. Intell. Transp. Syst..

[21] Lee Y., Yang J.H., Feron E., Kulkarni V. (2003). Development of a performance-based approach for a rear-end collision warning and avoidance system for automobiles. Proceedings of the IEEE IV2003: Intelligent Vehicles Symposium, Columbus, OH, USA, 9–11 June 2003.

[22] Rife J., Pervan B. (2012). Overbounding revisited: Discrete Error-Distribution modeling for Safety-Critical GPS navigation. IEEE Trans. Aerosp. Electron. Syst..

[23] Xiang X., Qin W., Xiang B. (2014). Research on a DSRC-Based Rear-End Collision Warning Model. IEEE Trans. Intell. Transp. Syst..

[24] Dion F., Rakha H., Kang Y.S. (2004). Comparison of delay estimates at under-saturated and over-saturated pre-timed signalized intersections. Transp. Res. Part B-Methodol..

[25] Zhang H., Sun J., Shao H. (2011). Revision of HCM delay model based on Bayesian inference. Comput. Eng..

[26] Chen Y.-L., Shen K.-Y., Wang S.-C. (2013). Forward collision warning system considering both time-to-collision and safety braking distance. Int. J. Veh. Saf..

[27] Tachet R., Santi P., Sobolevsky S., Reyes-Castro L.I., Frazzoli E., Helbing D., Ratti C. (2016). Revisiting street intersections using Slot-Based systems. PLoS ONE.

